# HPRNet: a hierarchical pyramidal residual network for ECG arrhythmia classification

**DOI:** 10.3389/fphys.2026.1800941

**Published:** 2026-05-08

**Authors:** Jiayan Huang, Miaomiao Huang, Hanling Zheng, Yongyi Xiao, Yolanda Bolea, Antoni Grau, Shaoye Luo

**Affiliations:** 1 Department of Systems Engineering, Automation and Industrial Informatics, Polytechnic University of Catalonia, Barcelona, Spain; 2 College of Computer and Data Science, Putian University, Putian, China; 3 College of Artificial Inteligence, Putian University, Putian, China

**Keywords:** deep learning, ECG arrhythmia classification, hierarchical pyramidal residual network, model pruning optimization, multi-scale feature learning

## Abstract

Accurate classification of electrocardiogram (ECG) signals plays a critical role in the automated diagnosis of cardiac arrhythmias. However, ECG recordings are often non-stationary and susceptible to various types of noise, which makes robust feature extraction challenging for many existing deep learning models. To address these challenges, this paper proposes a hierarchical pyramidal residual network (HPRNet) for ECG arrhythmia classification. HPRNet incorporates a hierarchical pyramidal REB-based backbone (HRB) to capture multi-scale morphological characteristics of ECG signals. In the HRB, the temporal resolution is progressively reduced while the number of feature channels is gradually increased, allowing the network to effectively learn multi-scale ECG representations. Furthermore, a multi-level pruning optimization (MLPO) strategy is incorporated, including network-level pruning and block-level pruning, to reduce redundant parameters and improve computational efficiency while preserving classification capability. Experiments on two public benchmark datasets show that HPRNet achieves superior performance compared with five representative methods on MIT-BIH, reaching an F1-score of 92.05%, while obtaining 91.98% on the INCART binary classification task with an average inference latency of 0.031 s. Moreover, visualization analysis highlights the intrinsic difficulty of distinguishing challenging beat classes, and ablation studies confirm the effectiveness of the proposed HRB and MLPO. These findings support the robustness of HPRNet for automated arrhythmia classification. The source code is publicly available at: https://github.com/jyanhuang/HPRNet-ECG.

## 1 Introduction

As a widely adopted non-invasive diagnostic modality, the electrocardiogram (ECG) provides rapid, lowcost, and reproducible measurements of cardiac electrical activity Berkaya et al. (2018). Its waveform patterns reflect instantaneous cardiac electrophysiological states, making ECG indispensable for early detection, risk stratification, and longitudinal monitoring of cardiovascular diseases (CVDs), particularly arrhythmias Wang et al. (2022a). However, early interpretation of ECG signals largely relied on manual examination and physician clinical experience, which could introduce subjectivity in waveform assessment and inter-observer variability. Moreover, ECG waveforms associated with different arrhythmia types often exhibit subtle morphological differences and are susceptible to various noise sources Wang et al. (2022b); Li et al. (2025b), posing significant challenges for reliable and accurate arrhythmia classification. Consequently, automated ECG signal classification has attracted extensive attention as a promising approach to reduce subjectivity in waveform interpretation and improve analysis consistency Ansari et al. (2023); Peng et al. (2024).

Existing ECG-based arrhythmia classification methods can be broadly categorized into traditional machine learning (ML)-based and deep learning (DL)-based methods. Representative ML-based methods include Random Forest (RF) Kropf et al. (2017) and Support Vector Machine (SVM) Adilakshmi (2024). These approaches typically rely on manual feature extraction and selection, which require substantial domain expertise to design effective features, thereby limiting their scalability and classification performance Marzog and Abd (2022). In contrast, DL-based methods, including convolutional neural networks (CNNs) Hannun et al. (2019), recurrent neural networks (RNNs), such as Long-Short-Term Memory (LSTM) Li et al. (2025a), and Transformer-based models Zhang et al. (2022), have demonstrated strong capabilities in ECG analysis. For instance, Zhu et al. (2020) proposed a deep learning framework for realtime multilabel diagnosis of heart rhythm and conduction abnormalities, while Jin et al. (2024) developed an interpretable DL-based model for detecting six common arrhythmias. Despite their promising performance, further improvements largely depend on enhancing the quality of deep feature representations rather than simply increasing network depth or model complexity. In this context, residual learning Choi et al. (2024) provides an effective mechanism for stabilizing deep network training by facilitating gradient propagation and alleviating the degradation problem, thereby improving feature extraction capability when the network architecture is properly designed. However, conventional residual architectures typically rely on straightforward stacking of residual blocks, which may result in excessive parameter growth and redundant computational overhead when applied to ECG signal analysis.

Therefore, to achieve stable training and effective feature extraction under complex ECG conditions, we propose a hierarchical pyramidal residual network (HPRNet) for ECG arrhythmia classification. Unlike conventional residual architectures, HPRNet adopts a modular and hierarchical residual design tailored to ECG signals, enabling the progressive extraction of discriminative temporal and morphological features directly from raw ECG data without requiring explicit denoising preprocessing. Furthermore, to alleviate the computational burden typically associated with hierarchical residual models, a Multi-Level Pruning Optimization (MLPO) strategy is introduced to control model complexity while maintaining classification capability, thereby enabling efficient and practical ECG-based arrhythmia analysis.

The main contributions of this paper are summarized as follows:

1. We propose a hierarchical pyramidal residual network (termed HPRNet) for multi-class ECG arrhythmia classification. HPRNet integrates convolutional feature extraction with a modular hierarchical residual architecture, enabling end-to-end representation learning directly from raw ECG signals without requiring explicit denoising preprocessing.
2. A hierarchical REB-based backbone (HRB) is designed using residual extraction blocks (REBs). Through channel expansion and same-channel downsampling transitions, the HRB progressively enlarges the temporal receptive field while maintaining stable feature propagation, enabling effective modeling of ECG waveform morphology and long-range rhythm dependencies.
3. To improve computational efficiency, a multi-level pruning optimization (MLPO) strategy is introduced. This strategy jointly performs network-level pruning to remove redundant parameters and block-level pruning to control the complexity of REBs, thereby reducing model size and computational overhead.
4. Extensive experiments on two public ECG benchmark datasets (MIT-BIH and INCART) demonstrate the effectiveness of the proposed HPRNet, achieving competitive classification performance while maintaining favorable computational efficiency. Furthermore, visualization analyses are performed to interpret the hierarchical feature representation behavior of HPRNet and to further analyze the challenging characteristics of S and F heartbeat categories.

The remainder of this paper is organized as follows. Section 2 reviews related work on ECG signal classification. Section 3 introduces the proposed method. Section 4 presents experimental results and ablation studies, as well as the limitations of the proposed method. Section 5 concludes the paper and discusses future research directions.

## 2 Related works

### 2.1 ECG signal concept and arrhythmia categories

**Figure 1:**
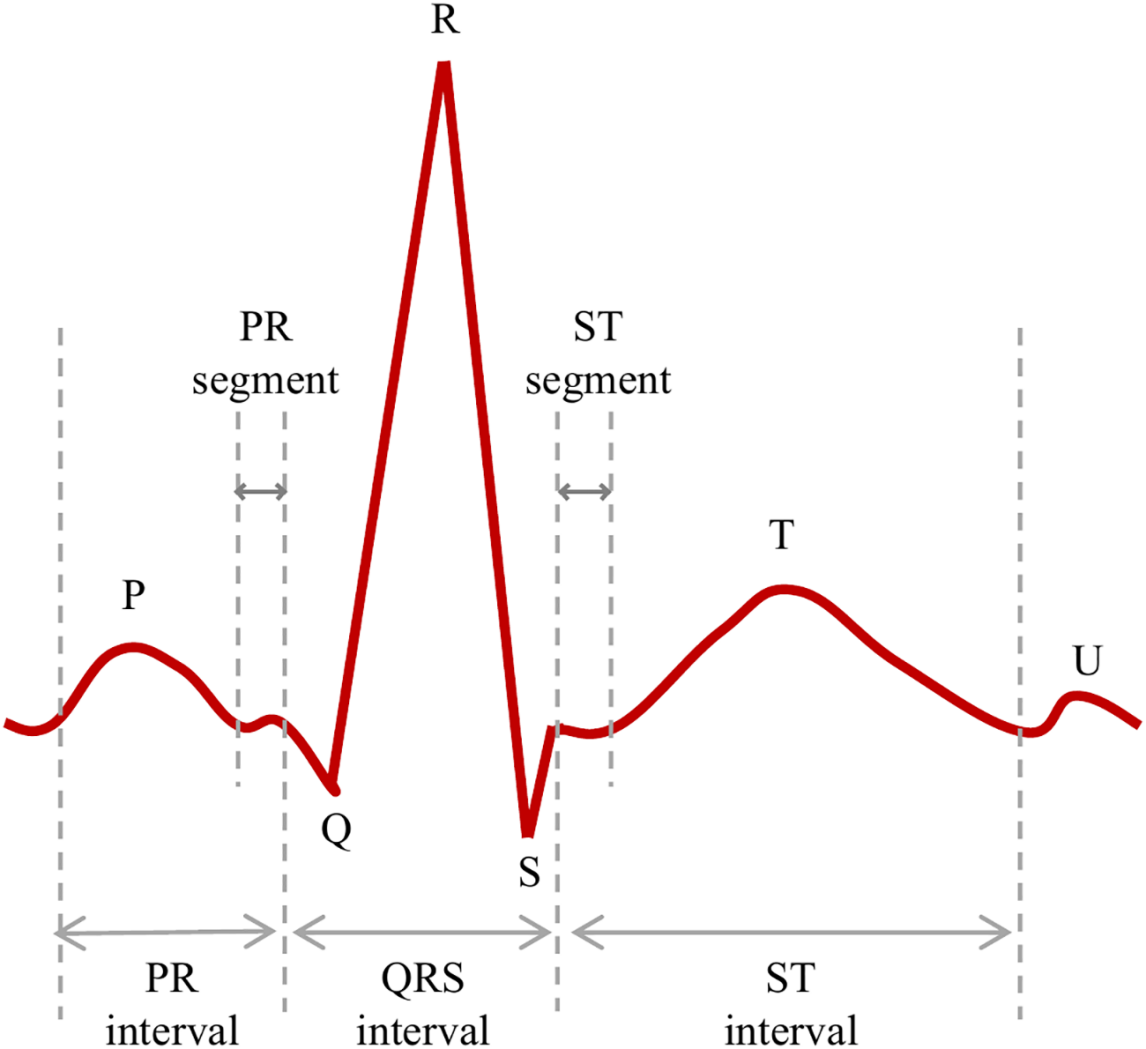
Morphological structure of a normal ECG signal.

The electrocardiogram (ECG) reflects the physiological and electrical status of the heart and provides essential information for cardiac assessment Abdulla and Al-Ani (2020). On the one hand, temporal intervals derived from ECG waveforms can assist clinicians in determining whether cardiac electrical activity is regular or irregular, as well as abnormally fast or slow. On the other hand, the amplitude and morphology of ECG signals can help indicate whether specific cardiac chambers are enlarged or under excessive workload. As shown in **Figure 1**, a normal ECG heartbeat consists of several characteristic components, including atrial depolarization (P wave), ventricular depolarization (QRS complex), and ventricular repolarization (T wave).

Abnormal cardiac electrical activity involving disturbed impulse generation or propagation is commonly referred to as arrhythmia and can be reflected in ECG signals. Representative arrhythmia types and abnormal ECG patterns commonly analyzed in ECG studies include supraventricular arrhythmias [e.g., Atrial Fibrillation (AF) and Atrial Flutter (AFL)], ventricular arrhythmias [e.g., Ventricular Fibrillation (VF) and Ventricular Flutter (VFL)], conduction abnormalities [e.g., Left Bundle Branch Block (LBBB) and Right Bundle Branch Block (RBBB)], Premature Atrial Contractions (PAC), and Premature Ventricular Contractions (PVC), Ventricular Escape Beats (VEB), and Paced Beats (PB). These ECG patterns are explicitly annotated in benchmark ECG datasets and are widely adopted in ECG classification research Abdulla and Al-Ani (2020); Özal Yildirim (2018).

### 2.2 Residual networks for ECG signal classification

Residual learning has been widely adopted for ECG classification and arrhythmia detection, particularly when deeper convolutional architectures are required to capture subtle morphological variations and multiscale temporal patterns in ECG signals. Zhang et al. (2023) presented a multi-class arrhythmia classification method based on a converted multi-scale residual neural network combined with multichannel data fusion. In their approach, extracted ECG features were transformed into a two-dimensional image representation for subsequent processing; however, such a conversion from one-dimensional signals to two-dimensional representations may introduce potential information loss. Khan et al. (2023) proposed an ECG classification model based on residual networks and demonstrated that ResNet outperforms conventional CNNs in deep feature extraction. Qi et al. (2024) proposed a Hybrid Residual Network (Hybrid ResNet) for ECG arrhythmia detection and classification, in which multiple convolutional operations are integrated, potentially increasing the architectural complexity of the network. Liu et al. (2025) proposed a spatio-temporal attention residual network that integrates residual learning with spatio-temporal attention and LSTM modules to model complex dependencies in ECG signals for multi-label classification tasks. Although residual learning effectively facilitates the training of deep networks, most existing studies primarily focus on improving feature representation capability, while comparatively less attention is paid to controlling parameter redundancy within residual blocks. Motivated by this observation, the proposed HPRNet incorporates a pruning strategy directly into the residual extraction blocks to reduce parameter redundancy while preserving the strong feature extraction ability of residual learning.

### 2.3 Model pruning strategies

The goal of ECG classification algorithms is practical clinical deployment, which imposes strict constraints on model size and computational complexity. Pruning can lower computational cost by eliminating redundant individual weights from the model. Han et al. (2015) adopted magnitudebased pruning by removing weights below a predefined threshold, followed by fine-tuning and regularization using L1 and L2 norms to recover performance. However, such approaches typically rely on post-pruning retraining, which may increase computational cost and limits scalability. Ashkboos et al. (2024) proposed SliceGPT, which reduces model complexity by removing rows and columns in weight matrices to shrink the embedding dimension while maintaining performance. In addition, Ma et al. (2023) investigated a gradient-based structured pruning strategy that selectively removes non-critical coupled structures, achieving effective acceleration while preserving the core functionality of the model. In this work, a non-structured pruning strategy is applied at both the network-level and within residual extraction blocks (block-level) to control parameter redundancy while maintaining classification performance.

## 3 The proposed method

### 3.1 Overall structure of HPRNet

ECG signals contain both local morphological patterns and long-term rhythm variations, requiring arrhythmia classification models to capture both local waveform characteristics and global rhythm information Liu et al. (2025). Therefore, we propose a hierarchical pyramidal residual network (HPRNet) for multi-class ECG arrhythmia classification. Specifically, along the depth direction, HPRNet progressively learns hierarchical ECG representations through multiple residual layers. Each residual layer is composed of several residual extraction blocks (REBs). This structure facilitates stable information and gradient propagation while enabling the model to capture ECG features at multiple temporal scales. However, as the network depth increases, the parameter size grows rapidly, leading to higher training complexity and computational cost. To address this issue, HPRNet introduces a multi-level pruning strategy that compresses redundant connections at both the network-level and the block-level, thereby reducing model complexity while preserving representational capacity.

**Figure 2:**
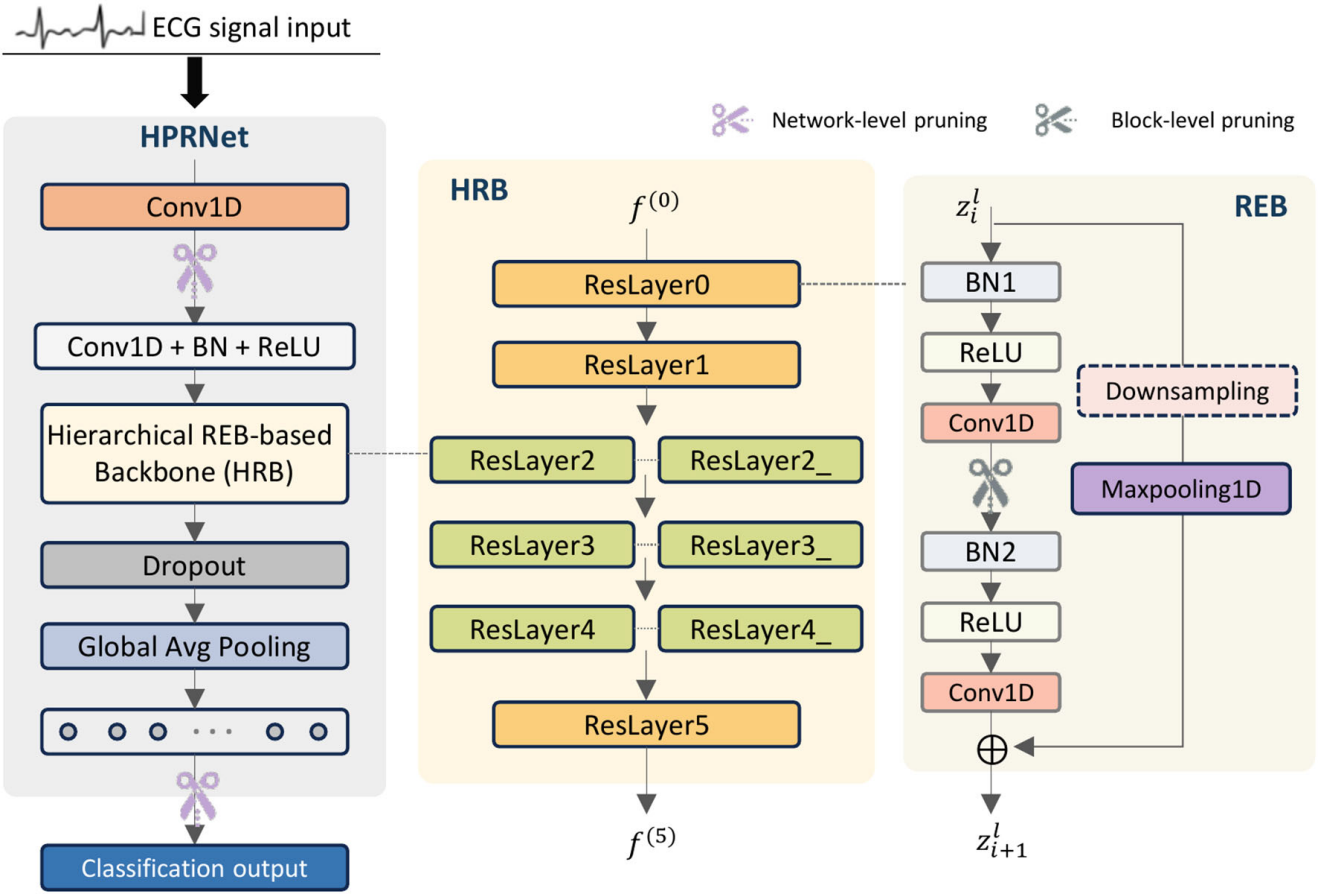
Structure of the proposed HPRNet.

Specifically, as shown in **Figure 2** and **Table 1**, let the input ECG signal be $x\in {\mathbb{R}}^{1\times L}$, where *L* denotes the length of the ECG segment. HPRNet first extracts initial temporal features using two one-dimensional convolutions Conv(·), batch normalization BN(·), and the ReLU activation function *δ*(·), which can be formulated as Equation 1:

**Table 1:** Layer-wise architecture of the proposed HPRNet network.

Stage	Layer/Module	Kernel size	Stride	Channels (In→Out)	Output size
Input	ECG beat input	–	–	1	1 × 128
Front-end	Conv1D	17	1	1 → 32	32 × 128
Front-end	Conv1D	17	1	32 → 32	32 × 128
Front-end	BatchNorm1D	–	–	32	32 × 128
Front-end	ReLU	–	–	32	32 × 128
HRB	ResLayer0	17	1	32 → 64	64 × 128
HRB	ResLayer1	17	2	64 → 64	64 × 64
HRB	ResLayer2	17	2	64 → 128	128 × 32
HRB	ResLayer2_	17	2	128 → 128	128 × 16
HRB	ResLayer3	17	2	128 → 256	256 × 8
HRB	ResLayer3_	17	2	256 → 256	256 × 4
HRB	ResLayer4	17	2	256 → 512	512 × 2
HRB	ResLayer4_	17	2	512 → 512	512 × 1
HRB	ResLayer5	17	2	512 → 1024	1024 × 1
Classifier	Dropout	–	–	1024	1024 × 1
Classifier	Global AvgPool1D	–	–	1024	1024 × 1
Classifier	Fully Connected	–	–	1024 → *C*	*C*

(1) $$f_{0}=\delta \left(\text{BN}\left(\text{Conv}2\left(\text{Conv}1\left(x\right)\right)\right)\right).$$

The initial feature *f*_0_ is then fed into a Hierarchical REB-Based Backbone (HRB) network ℋ(·) to progressively learn hierarchical ECG representations, which can be defined as Equation 2:

(2) $$f_{\text{deep}}=ℋ\left(f_{0}\right).$$

After obtaining the deep feature map, dropout is applied to mitigate overfitting. Global average pooling (GAP) is then used to aggregate temporal features, followed by a fully connected layer to generate the classification logits, as shown in Equation 3:

(3) $$y=W_{f}\cdot \text{GAP}(f_{\text{deep}})+b_{f},$$

where *W_f_* and *b_f_* denote the weight and bias of the fully connected layer, respectively. Finally, the predicted class probabilities are obtained using the Softmax function according to Equation 4:

(4) $$p_{i}=\frac{e^{y_{i}}}{\sum_{j=1}^{C}e^{y_{j}}},\;i=1,2,\ldots ,C,$$

where *C* denotes the number of arrhythmia classes.

### 3.2 Hierarchical REB-based backbone

To progressively extract discriminative ECG representations, we design a hierarchical pyramidal REBbased backbone (HRB) for HPRNet. As shown in **Figure 2**, the HRB backbone is organized as a hierarchical stack of nine residual layers (ResLayers), each composed of multiple residual extraction blocks (REBs). The numbers of REBs in the nine ResLayers are [3, 4, 4, 4, 4, 4, 4, 4, 3], where the shallow and final stages contain fewer blocks, while the intermediate stages adopt more REBs to strengthen feature representation. Between adjacent ResLayers, strided convolutions are used to progressively reduce the temporal resolution while expanding the channel dimension, forming a hierarchical pyramidal representation. In addition, several intermediate stages employ same-channel downsampling layers to further enlarge the receptive field without increasing model complexity. This design allows the backbone to progressively capture high-level semantic information and long-range temporal dependencies in ECG signals.

Specifically, at the inter-layer level, the HRB propagates features across residual layers. Let *f* ^(0)^ = *f*_0_ denote the input feature to the backbone. The output of the *l*-th ResLayer is defined as Equation 5:

(5) $$f^{\left(l+1\right)}={ℛ}_{l}\left(f^{\left(l\right)}\right),\;l=0,1,\ldots ,L-1.$$

At the intra-layer level, each residual layer refines features through a cascade of residual extraction blocks (REBs), which can be expressed as Equation 6:

(6) $${ℛ}_{l}\left(x\right)={ℬ}_{l,N_{l}}\left({ℬ}_{l,N_{l}-1}\left(\cdots {ℬ}_{l,1}\left(x\right)\right)\right),$$

where ${ℬ}_{l,k}\left(\cdot \right)$ denotes the *k*-th REB in the *l*-th residual layer and *N_l_* represents the number of REBs in that layer.

### 3.3 Residual extraction blocks

In this work, multiple residual extraction blocks (REBs) are employed to construct the HRB for capturing subtle discriminative patterns in ECG signals. As illustrated in **Figure 2**, each REB consists of a main branch and a skip branch. The main branch adopts a pre-activation structure and performs two BN-ReLU-Conv1D operations to progressively extract deeper temporal features. Large-kernel convolution with a kernel size of 17 and padding of 8 is used to maintain temporal resolution while ensuring dimensional consistency for residual addition. Such a kernel size is well aligned with the temporal scale of key ECG morphological components, such as the QRS complex and short-term rhythm variations. For the skip branch, a 1×1 convolution with stride is first applied to downsample the input feature and match the channel dimension of the main branch output, followed by a max-pooling operation to preserve salient temporal responses. Compared with traditional residual blocks using identity or linear projection shortcuts, the proposed pooling-based skip connection introduces a nonlinear selection mechanism that suppresses low-activation responses while retaining important temporal features.

Let the initial input be $z_{0}^{\left(0\right)}=f^{\left(0\right)}$. The output of the *i*-th REB in the *l*-th ResLayer can be expressed as Equation 7:

(7) $$z_{i+1}^{\left(l\right)}={ℱ}_{p}\left(z_{i}^{\left(l\right)}\right)+P\left(D\left(z_{i}^{\left(l\right)}\right)\right),$$

where ${ℱ}_{p}\left(\cdot \right)$ denotes the pruned residual mapping, D(·) represents the downsampling operation (1 × 1 convolution), and P(·) denotes the max pooling operation. This design enables the REB to simultaneously enhance temporal feature extraction while reducing parameter redundancy, thereby improving the efficiency and representational capability of the backbone network.

### 3.4 Multi-level pruning optimization

Although stacked convolutions are capable of learning rich feature representations, they may also introduce parameter redundancy and increase training complexity in deep hierarchical architectures. To this end, a multi-level pruning optimization (MLPO) strategy is proposed to remove redundant weights while preserving discriminative ECG features.

Specifically, network-level, pruning is applied to the first convolutional layer and the final fully connected layer of HPRNet to remove low-contribution weights in the feature extraction and classification stages. Block-level pruning is embedded into each residual extraction block (REB) by pruning the weights of the first convolution in the main branch, which suppresses redundant intermediate representations introduced by stacked convolutions. The pruning process follows magnitude-based pruning, where weights with small L1-norm magnitudes are removed according to a predefined pruning ratio.

## 4 Experimental results

This section first introduces the datasets, evaluation metrics, and experimental setup. Then, the classification performance of the proposed method is compared with that of five representative ECG classification methods. Finally, visualization analyses are provided to interpret the hierarchical feature representation behavior of the proposed model, and ablation studies are conducted to investigate the effects of the key designs in HPRNet, including HRB, REBs, and MLPO.

### 4.1 ECG signal datasets

We used the MIT-BIH Moody and Mark (2001) and INCART Goldberger et al. (2000) datasets to train the network and comprehensively evaluate the classification performance.

MIT-BIH: The MIT-BIH Arrhythmia Database is a widely used public ECG dataset for arrhythmia analysis. It contains 48 half-hour two-lead ECG recordings collected from 47 subjects, comprising more than 110,000 annotated heartbeats labeled by cardiologists. The ECG signals are sampled at 360 Hz with 11-bit resolution and include 15 types of arrhythmia annotations.

INCART: The INCART Arrhythmia Database contains 12-lead ECG recordings with various types of arrhythmias. The dataset provides more diverse and complex ECG patterns, making it suitable for evaluating the generalization ability of classification models. The ECG signals are sampled at 257 Hz.

**Figure 3:**
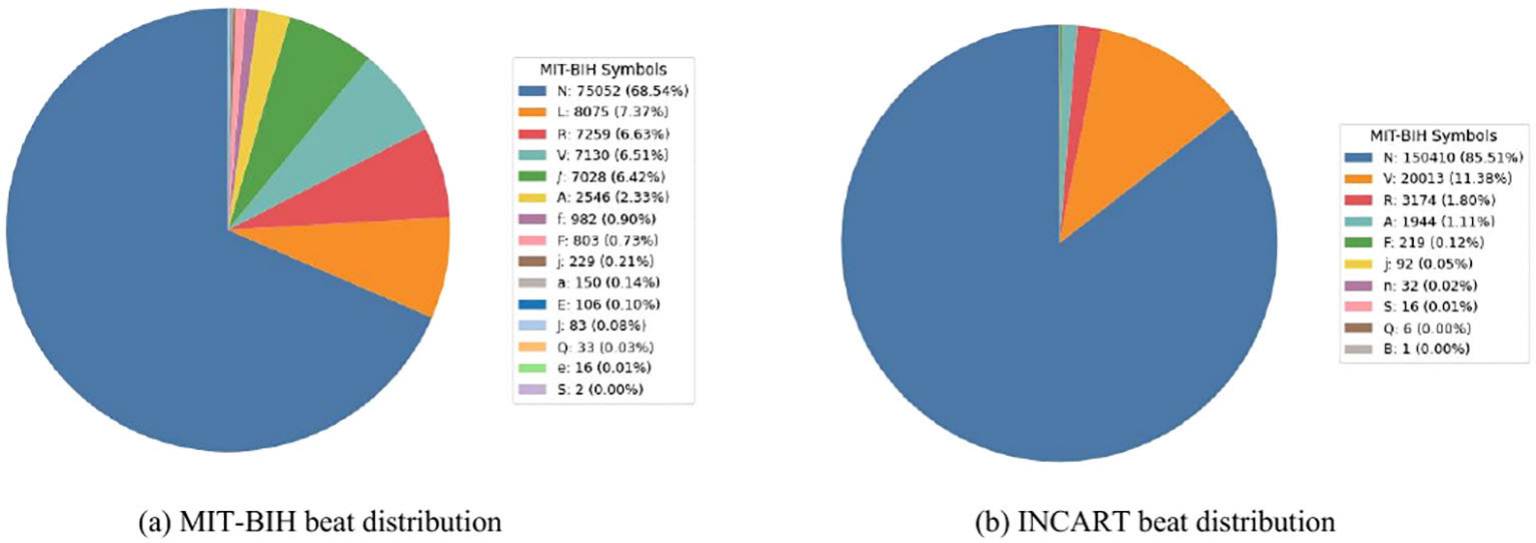
Category distributions of the MIT-BIH and INCART datasets based on the original annotation labels. **(a)** MIT-BIH beat distribution; **(b)** INCART beat distribution.

**Figure 4:**
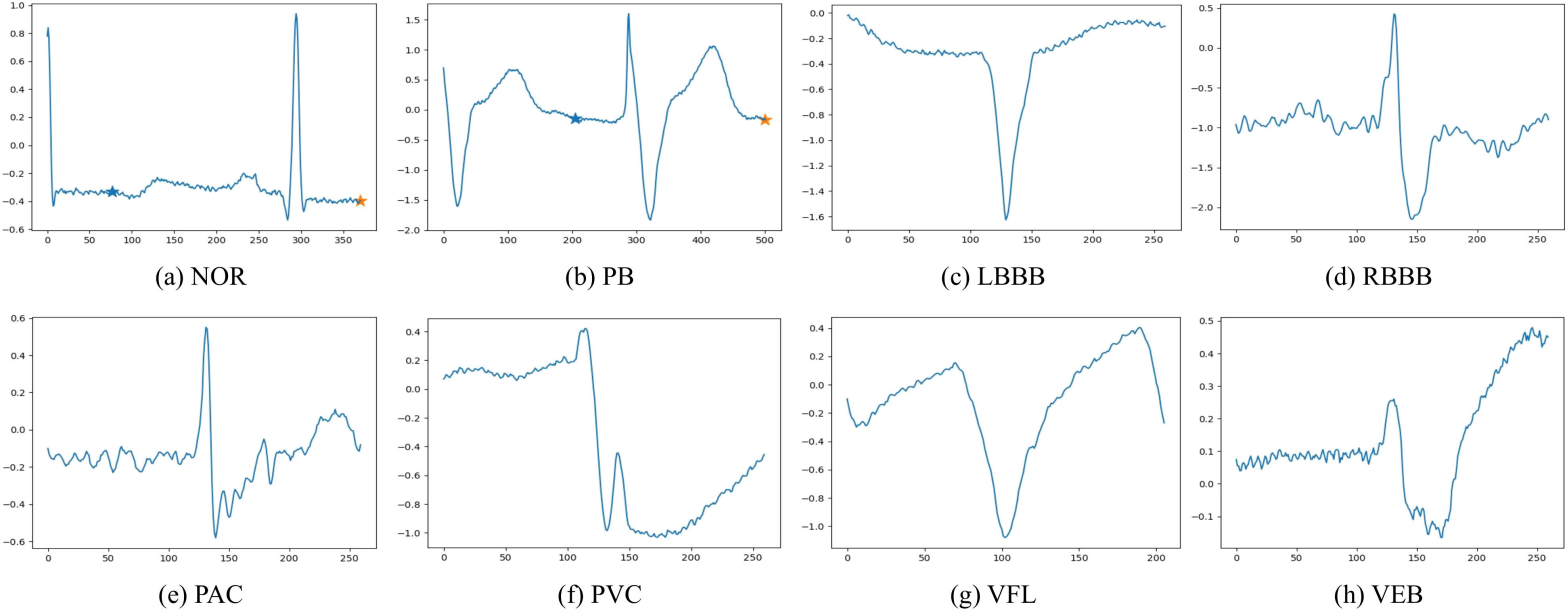
Representative ECG beats of different heartbeat types from the MIT-BIH arrhythmia dataset. **(a)** Normal beat (NOR); **(b)** Paced beat (PB); **(c)** Left bundle branch block beat (LBBB); **(d)** Right bundle branch block beat (RBBB); **(e)** Premature atrial contraction (PAC); **(f)** Premature ventricular contraction (PVC); **(g)** Ventricular flutter (VFL); **(h)** Ventricular escape beat (VEB).

**Figure 5:**
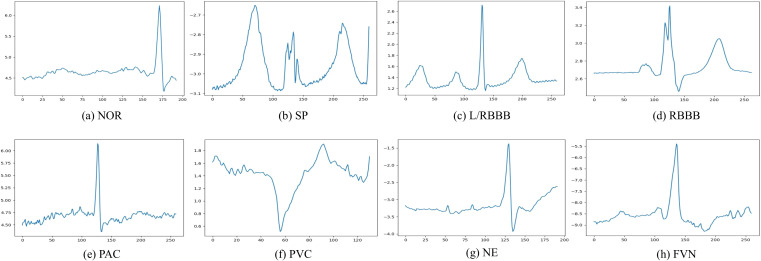
Representative ECG beats of different heartbeat types from the INCART dataset. **(a)** Normal beat (NOR); **(b)** Supraventricular premature beat (SP); **(c)** Left/right bundle branch block beat (L/RBBB); **(d)** Right bundle branch block beat (RBBB); **(e)** Premature atrial contraction (PAC); **(f)** Premature ventricular contraction (PVC); **(g)** Nodal escape beat (NE); **(h)** Fusion of ventricular and normal beat (FVN).

**Figure 3** illustrates the category distributions of the MIT-BIH and INCART datasets. It can be observed that the INCART dataset exhibits a more imbalanced distribution compared with MIT-BIH. In addition, **Figures 4**, **5** present representative ECG signal samples from the MIT-BIH and INCART datasets, respectively. For both datasets, only the MLII lead was used in this study.

### 4.2 Evaluation indicators

To comprehensively evaluate the performance of different methods, Accuracy, Precision, Recall, and F1-score were used to measure the classification performance for each class Opitz (2024). In addition, confusion matrices were provided to analyze the class-wise prediction behavior. These metrics are defined as Equations 8–11:

(8) $$Accuracy=\frac{TP+TN}{TP+TN+FP+FN}$$

(9) $$Precision=\frac{TP}{TP+FP}$$

(10) $$Recall=\frac{TP}{TP+FN}$$

(11) $$F1-score=2\cdot \frac{Precision\cdot Recall}{Precision+Recall}$$

where *TP* denotes true positives, *TN* denotes true negatives, *FP* denotes false positives, and *FN* denotes false negatives.

Furthermore, the number of parameters (Params), average inference time (Time), and million floatingpoint operations (MFLOPs) were used to evaluate the model complexity and computational cost. Specifically, Params reflects the overall model size, Time denotes the average inference latency per ECG segment under identical experimental settings, and MFLOPs quantify the number of floating-point operations required for a single inference.

### 4.3 Experimental setup

To comprehensively evaluate the model performance, multiple classification tasks were designed on the MIT-BIH and INCART datasets. For the MIT-BIH dataset, a four-class classification task was constructed according to the AAMI standard, as shown in **Table 2**. For the INCART dataset, both normal-abnormal binary classification and AAMI-like three-class classification tasks were designed to analyze the model performance under different task difficulties. During training, we used five-fold cross-validation to evaluate the classification performance.

**Table 2:** AAMI standard on MIT-BIH and AAMI-like standard on INCART, as well as the corresponding number of heart beats.

AAMI (-like)	Type description	MIT-BIH symbols	Number of beats	INCART symbols	Number of beats
N	Normal beat	N,L,R,e,j	90,631	N,R,j,n,B	153,709
S	Supraventricular ectopic beat	A,a,J,S	2,781	A,S	1,960
V	Ventricular ectopic beat	V, E	7,236	V	200,232
F	Fusion beat	F	803	N/A	N/A
Q	Unknown beat	/,f, Q	8,043	Q	6
Total	–	N,L,R,e,j,A,a,J,S,V, E, F,/,f,Q	109,494	N,L,R,j,n,B,A,S,V,F,Q	175,907

All experiments were conducted on a workstation equipped with an Intel Core i9-14900HX CPU, 32 GB RAM, and an NVIDIA RTX 5070 Ti GPU. The proposed HPRNet model was implemented using Python 3.10 and the PyTorch deep learning framework. During training, the Adam optimizer was adopted with an initial learning rate of 0.001 and a batch size of 128. The maximum number of training epochs was set to 30, and the learning rate was adaptively adjusted during training.

### 4.4 Classification results

#### 4.4.1 Results on MIT-BIH

To comprehensively evaluate the proposed HPRNet, we compare it with several representative ECG heartbeat classification methods, including Ensemble_SVM Mondéjar-Guerra et al. (2019), SE-ECGNet Chen et al. (2020), ECGTransForm Alamr and Artoli (2023), SRT Wu et al. (2024), and LightweightCNN Thota et al. (2025). For a fair comparison, all baseline models were implemented following the original hyperparameter settings reported in their respective publications. The detailed experimental configurations are summarized in **Table 3**.

**Table 3:** The hyperparameter settings of different methods.

Methods	Framework	Optimizer	Epochs	Batch size	Learning_rate (initial)
Ensemble_SVM Mondéjar-Guerra et al. (2019)	Scikit-learn	Adam	60	128	1e-3
SE-ECGNet Chen et al. (2020)	PyTorch	Adam	256	64	1e-3
ECGTransForm Alamr and Artoli (2023)	PyTorch	Adam	60	128	1e-3
SRT Wu et al. (2024)	N/A	N/A	N/A	N/A	N/A
LightweightCNN Thota et al. (2025)	Keras	Adam	30	32	1e-3
ours	PyTorch	Adam	30	128	1e-3

**Figure 6:**
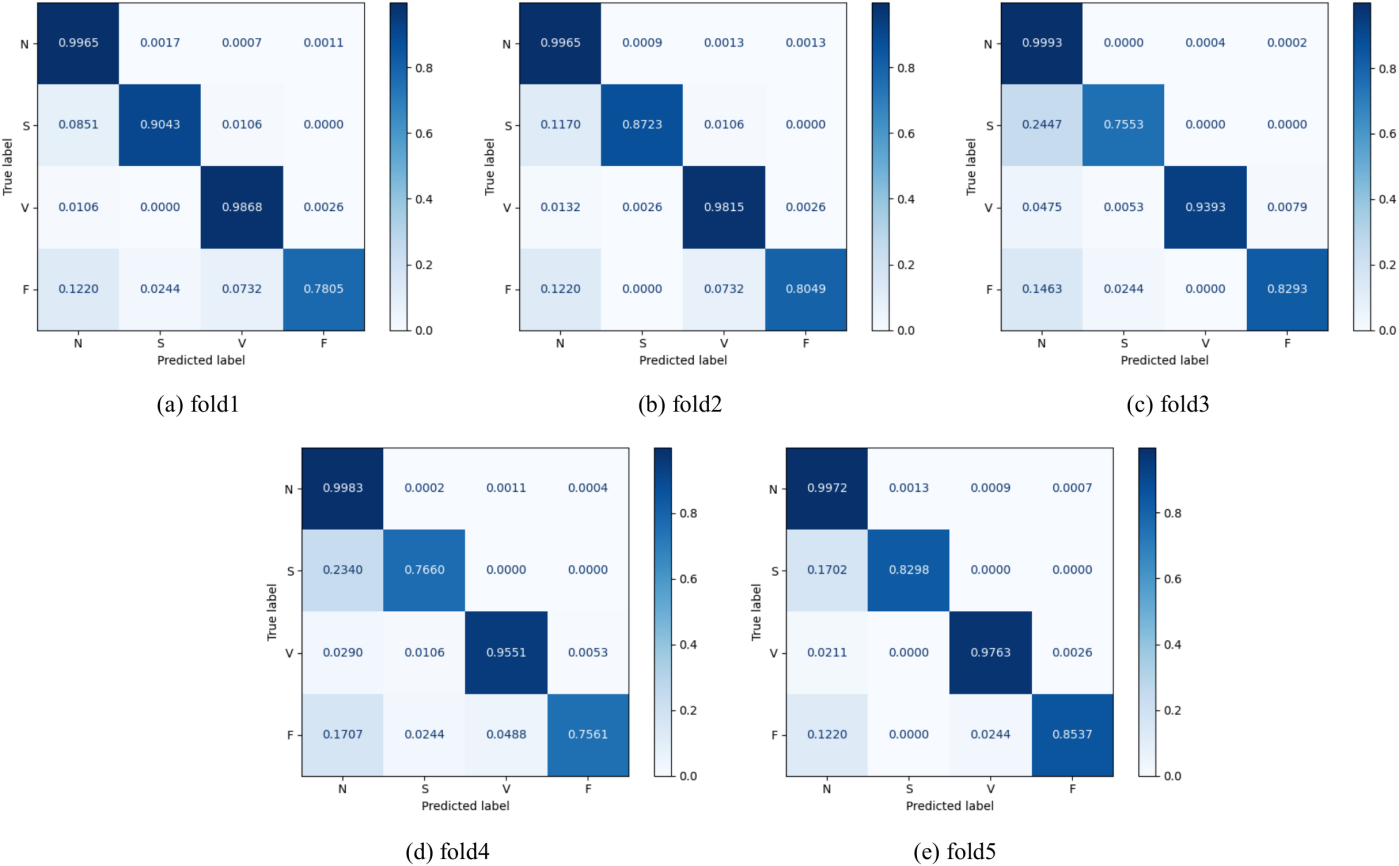
Confusion matrix of the HPRNet on MIT-BIH. **(a)** fold1; **(b)** fold2; **(c)** fold3; **(d)** fold4; **(e)** fold5. The abbreviations used in the confusion matrix follow the AAMI standard: N (Normal beat), S (Supraventricular ectopic beat), V (Ventricular ectopic beat), F (Fusion beat).

Specifically, five-fold cross-validation was conducted on the MIT-BIH dataset to evaluate the proposed HPRNet model. As shown in **Table 4**, HPRNet achieved an average accuracy of 98.97%, reflecting its strong overall classification capability. Meanwhile, the average precision, recall, and F1-score reached 94.63%, 89.89%, and 92.05%, respectively, demonstrating its balanced performance in terms of sensitivity and overall classification effectiveness. For class-wise results, *F*1*_N_* and *F*1*_V_* reached 99.53% and 97.58%, respectively, indicating that the model can effectively identify N and V heartbeats. By comparison, the lower values of *F*1*_S_* and *F*1*_F_* suggest that the classification of S and F heartbeats is more challenging. This phenomenon may be attributed to class imbalance, as the numbers of S and F samples are relatively small, leading to insufficient learning of minority-class features. In addition, these two types of heartbeats share certain morphological similarities with other categories, which further increases the difficulty of classification. This observation is also reflected in the confusion matrices shown in **Figure 6**, where most samples are concentrated along the main diagonal across all five folds, while misclassifications are more likely to occur in the S and F categories. Nevertheless, the small performance fluctuations across the five folds demonstrate that the proposed HPRNet has good classification performance, robustness, and stability.

**Table 4:** The results of HPRNet using five-fold cross-validation on MIT-BIH dataset.

Fold ID	Accuracy	Precision	Recall	F1*_N_*	F1*_S_*	F1*_V_*	F1*_F_*	F1*_avg_*
1	99.04	93.11	91.70	99.64	90.43	98.42	81.01	92.38
2	98.94	93.41	91.38	99.59	90.61	97.77	81.48	92.36
3	98.89	95.96	88.08	99.45	84.52	96.61	86.08	91.67
4	98.88	95.50	86.84	99.46	84.15	96.94	82.05	90.65
5	99.12	95.16	91.43	99.53	87.64	98.14	87.50	93.20
Average	98.97	94.63	89.89	99.53	87.47	97.58	83.62	92.05

**Table 5** further compares the proposed HPRNet with several representative ECG heartbeat classification methods on the MIT-BIH dataset in terms of both classification performance and computational efficiency. As shown in the table, HPRNet achieves the best overall classification performance, with an accuracy of 98.97%, precision of 94.63%, recall of 89.89%, and an F1-score of 92.05%. In terms of efficiency, although HPRNet does not have the smallest parameter count or MFLOPs, it achieved the lowest inference latency (0.0312 s). This suggests that the proposed model provides a favorable balance between classification accuracy and practical inference efficiency.

**Table 5:** The classification and efficiency comparisons of different methods on MIT-BIH dataset.

Models	Classification performance ↑				Efficiency ↓		
	Accuracy (%)	Precision (%)	Recall (%)	F1-score (%)	Params (#)	Latency (s)	MFLOPs
Ensemble_SVM Mondéjar-Guerra et al. (2019)	94.50	66.40	70.30	68.29	8,253,360	0.0362	**1.774**
SE-ECGNet Chen et al. (2020)	91.67	83.33	60.60	70.18	16,496,035	1.3504	1483.304
ECGTransFormAlamr and Artoli (2023)	96.34	88.29	89.53	88.84	**637,431**	0.0800	7.710
SRT Wu et al. (2024)	95.70	78.60	88.10	82.60	–	–	–
LightweightCNN Thota et al. (2025)	90.72	79.00	77.00	77.99	940,069	0.1641	193.917
ours	**98.97**	**94.63**	**89.89**	**92.05**	19,110,641	**0.0312**	713.142

↑ denotes the higher and the better, and ↓ denotes the lower the better.

The bold values indicate the best results among the compared methods.

#### 4.4.2 Results on INCART

**Figure 7:**
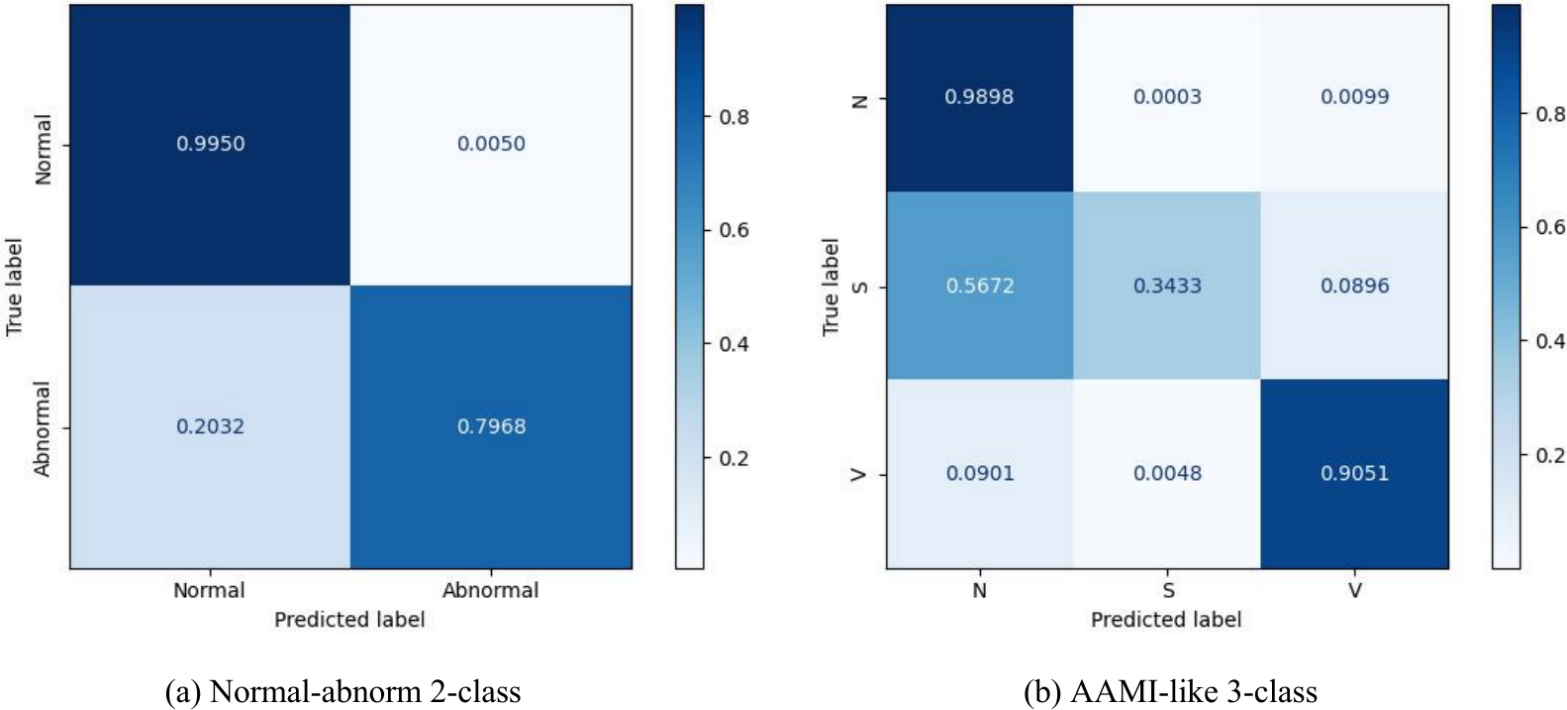
Confusion matrix of the HPRNet on INCART. **(a)** Normal-abnormal 2-class classification. **(b)** AAMI-like 3-class classification. The abbreviations used in the confusion matrix follow the AAMI-like standard: N (Normal beat), S (Supraventricular ectopic beat), V (Ventricular ectopic beat).

The experimental results of different classification tasks on the INCART dataset are summarized in **Table 6**, and the corresponding confusion matrices are illustrated in **Figure 7**. For the normal-abnormal binary classification task, the proposed model achieves an accuracy of 98.41%, with precision, recall, and F1-score of 94.51%, 89.59%, and 91.98%, respectively. As shown in **Figure 7A**, the model correctly identifies 99.50% of normal beats, while 79.68% of abnormal beats are accurately detected, demonstrating a good capability in distinguishing normal and abnormal heartbeats.

**Table 6:** The results of different classification tasks on INCART dataset.

Task types	Accuracy (%)	Precision (%)	Recall (%)	F1-score (%)
Binary-classification	98.41	94.51	89.59	91.98
AAMI three-classification	98.32	84.15	74.60	79.09

For the AAMI-like three-class classification task, the proposed method also achieves competitive performance, with an overall accuracy of 98.32%. As shown in **Figure 7B**, the model performs very well in identifying normal beats (98.98%) and ventricular ectopic beats (90.51%). In contrast, the recognition of S remains challenging, with a recall of only 34.33%. This limitation is mainly attributed to the severe class imbalance in the INCART dataset, where S beats are significantly underrepresented, as well as their morphological similarity to normal beats. Consequently, a large proportion of S beats are misclassified as normal beats, which degrades the overall performance in this category. Nevertheless, the proposed model still demonstrates discriminative ability and stable performance across different classification tasks on the INCART dataset.

### 4.5 Visualization analysis

To further investigate the hierarchical representation behavior of HPRNet, Gradient-weighted Class Activation Mapping (Grad-CAM) was employed to visualize the feature responses of different backbone layers.

#### 4.5.1 Hierarchical representation analysis

**Figure 8:**
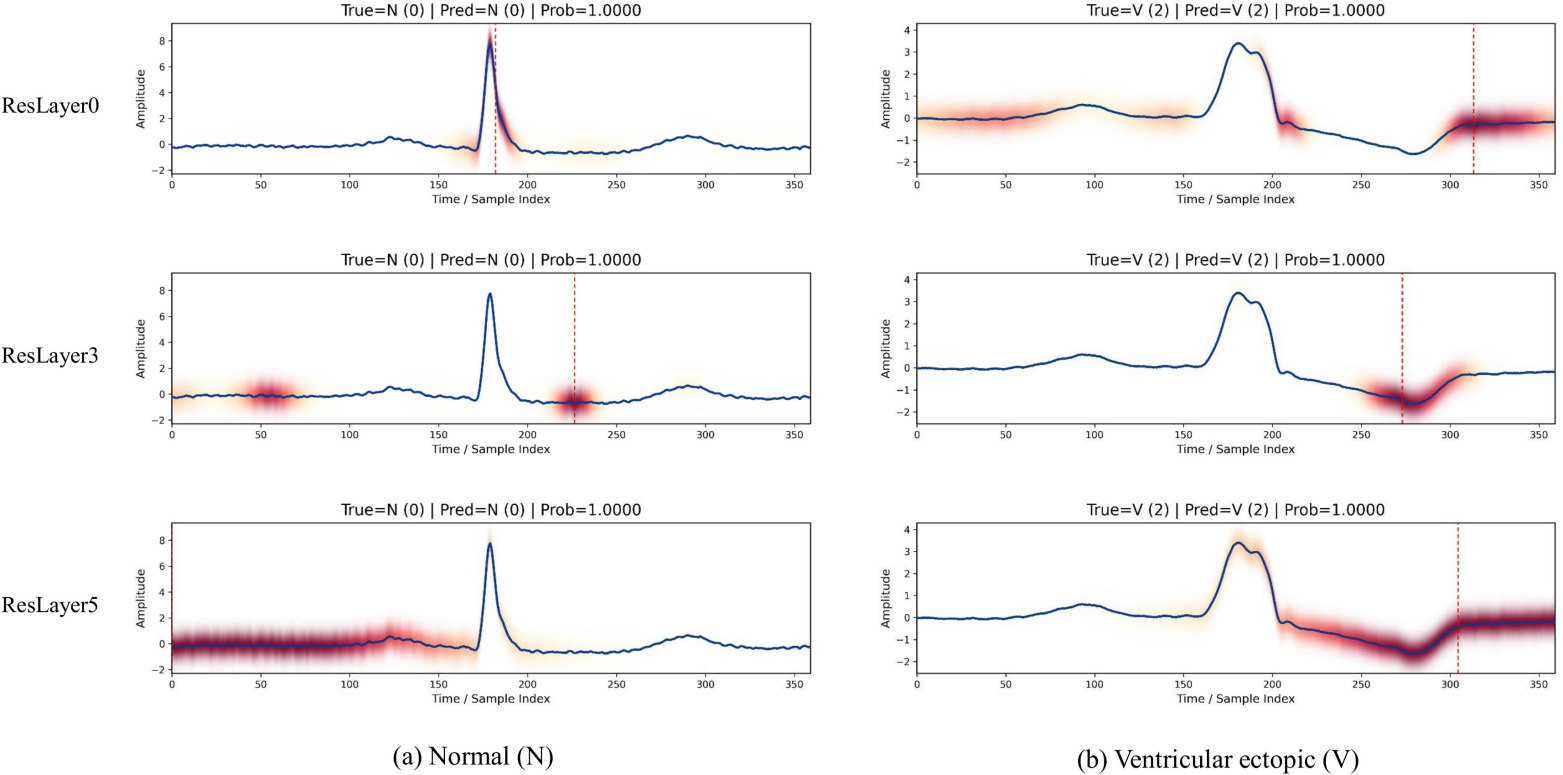
Grad-CAM visualizations of representative N and V heartbeat samples at shallow (ResLayer 0), intermediate (ResLayer 3), and deep (ResLayer 5) layers of HPRNet. **(a)** Normal beat (N). **(b)** Ventricular ectopic beat (V).

**Figure 8** presents the Grad-CAM responses of representative normal (N) and ventricular ectopic (V) beats at different layers of the proposed HPRNet. In the shallow layer, the network mainly focuses on prominent local waveform structures, particularly the QRS complex. As the depth increases, the receptive field gradually expands, and the model begins to capture broader morphological characteristics, including waveform regions related to the P wave and T wave. In deeper layers, the network learns more global heartbeat representations, highlighting discriminative regions associated with ventricular ectopic beats, such as abnormal ST segments and waveform distortions. These observations indicate that the proposed hierarchical pyramidal backbone effectively learns multi-level ECG representations, enabling the model to progressively extract discriminative features for arrhythmia classification.

#### 4.5.2 Analysis of challenging S and F categories

**Figure 9:**
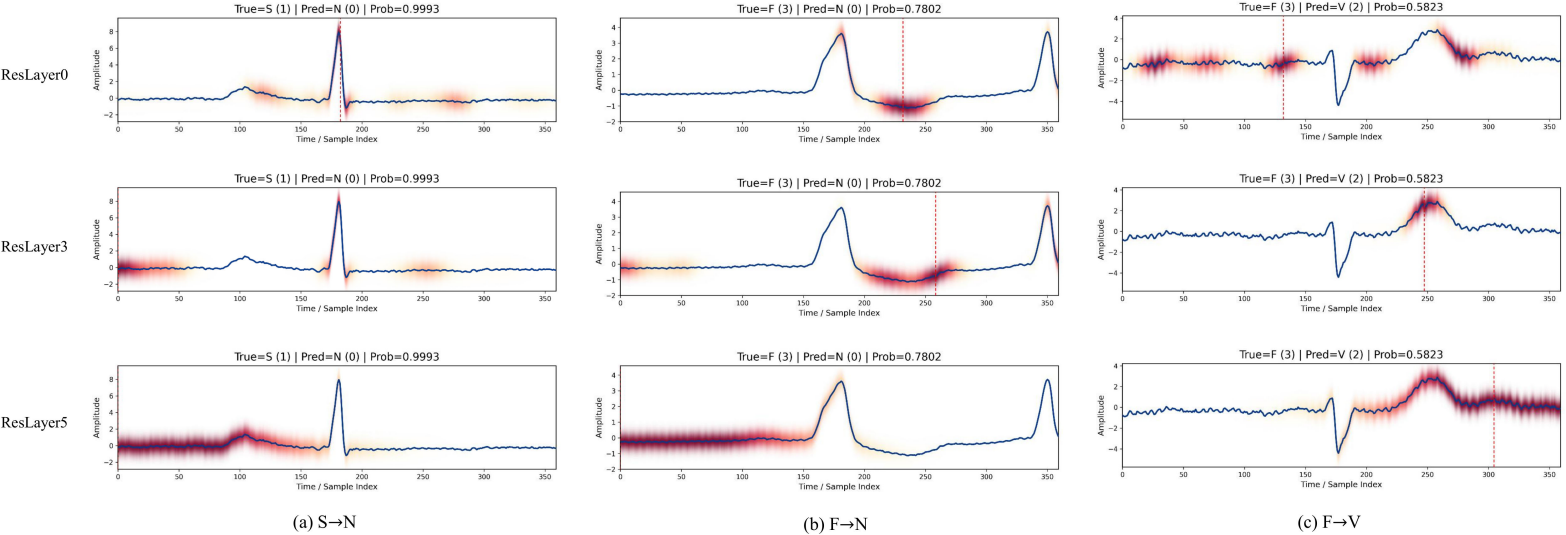
Grad-CAM visualizations of representative misclassified heartbeat samples across shallow (ResLayer 0), intermediate (ResLayer 3), and deep (ResLayer 5) layers of HPRNet. **(a)** S→N case. **(b)** F→N case. **(c)** F→V case.

**Figure 9** presents the Grad-CAM responses of different backbone layers for representative misclassified heartbeat samples. For the S→N case, the shallow layer mainly focuses on the QRS complex, while deeper layers gradually incorporate broader waveform context. However, due to the morphological similarity between S and N, the discriminative features remain insufficiently distinctive, resulting in misclassification. For the F→N and F→V cases, the activation maps show that the model progressively attends to abnormal waveform regions such as the ST segment and T wave. Nevertheless, the hybrid morphology of fusion beats shares characteristics with both normal and ventricular beats, thereby increasing the classification ambiguity. These observations indicate that the proposed hierarchical backbone captures multi-scale ECG representations, while also revealing the intrinsic difficulty in distinguishing S and F heartbeat categories.

### 4.6 Ablation studies

#### 4.6.1 Discussion on noise robustness

The proposed HPRNet adopts a hierarchical pyramidal residual architecture that facilitates stable feature learning while preserving essential ECG signal characteristics. Owing to this structural advantage, explicit signal denoising is not required as a mandatory preprocessing step. To further investigate this aspect, an additional experiment was conducted to evaluate the impact of ECG denoising on classification performance. Specifically, a representative wavelet-based denoising method was applied using the sym8 wavelet with five decomposition levels and soft-thresholding to suppress high-frequency noise Ádám et al. (2025). The corresponding classification results are summarized in **Table 7**. As shown in **Table 7**, only marginal differences are observed between the denoised and raw ECG inputs across all evaluation metrics, and the non-denoised setting even achieves slightly better performance. These results indicate that the proposed HPRNet can inherently learn noise-robust representations from raw ECG signals, thereby maintaining stable classification performance without additional denoising preprocessing.

**Table 7:** Comparisons of classification results with and without denoising preprocessing on MIT-BIH dataset.

Denoising preprocessing	Accuracy (%)	Precision (%)	Recall (%)	F1 score (%)
✓	98.86	92.55	90.17	91.15
×	98.97	94.63	89.89	92.05

#### 4.6.2 Discussion on the effect of HRB

To further evaluate the effectiveness of the proposed HRB in feature extraction, ablation experiments were conducted by replacing the HRB with standard convolutional layers and removing the residual skip-connections in REBs, respectively. As shown in **Table 8**, the standard CNN achieves an accuracy of only 74.79%, indicating that simple convolutional stacking is insufficient to capture discriminative ECG features. Removing residual skip-connections improves the accuracy to 94.25%, highlighting the importance of residual feature propagation for deep representation learning. This is consistent with previous findings on residual networks Ranganathan et al. (2025). By further incorporating the proposed residual extraction blocks (REBs) to construct the HRB backbone, the model further achieves 98.97% accuracy without increasing the number of parameters, demonstrating the effectiveness of REBs in enhancing feature representation and stabilizing deep network training.

**Table 8:** Impact of the HRB on the HPRNet Backbone.

Backbone configuration	Accuracy (%)	Params (#)
std. CNN	74.79	**19,005,998**
w/o skip-connections	94.25	19,110,641
HRB (ours)	**98.97**	19,110,641

The bold values indicate the best results among the compared configurations.

#### 4.6.3 Impact of the number of REBs

In this work, residual extraction blocks (REBs) are adopted as the basic building units of the HRB backbone to facilitate the extraction of deeper and more discriminative signal features. However, increasing the number of REBs may lead to a substantial growth in model parameters. Therefore, it is necessary to balance classification performance with model complexity. To investigate the impact of the number of REBs, a series of experiments were conducted using different REB configurations. **Table 9** reports the classification accuracy and parameter numbers of HPRNet with varying numbers of REBs in each ResLayer. As shown in **Table 9**, the configuration [3,4,4,4,4,4,4,4,3] achieves the highest classification accuracy while maintaining a moderate number of parameters, demonstrating a favorable trade-off between model performance and computational complexity.

**Table 9:** Trade-off between classification accuracy and parameter number of different REB configurations.

REBs number for each ResLayer	Accuracy (%)	Params (#)
[2,3,3,3,3,3,3,3,2]	98.63	**13,166,219**
[3,4,4,4,4,4,4,4,3]	**98.97**	19,110,641
[4,5,5,5,5,5,5,5,4]	98.84	25,055,063
[5,6,6,6,6,6,6,6,5]	98.67	30,999,485
[6,7,7,7,7,7,7,7,6]	98.64	36,943,907

The bold values indicate the best results among the compared configurations.

#### 4.6.4 Impact of the MLPO strategy

In this work, a multi-level pruning optimization (MLPO) strategy is adopted to compress HPRNet by jointly applying network-level pruning to both the first convolution and full-connected layers and block-level pruning to the REBs. To evaluate the effectiveness of different pruning strategies, ablation experiments were conducted by comparing models without pruning, with network-level pruning, with block-level pruning, and with the complete MLPO strategy. As shown in **Table 10**, both block-level and network-level pruning effectively reduce the number of model parameters while maintaining comparable classification performance, and their effects are complementary. The complete MLPO strategy achieves the best performance with the lowest model complexity, demonstrating that multi-level pruning can effectively improve computational efficiency while preserving robust arrhythmia classification capability.

**Table 10:** Performance comparisons of different pruning configurations for HPRNet.

Pruning configurations	Accuracy (%)	Precision (%)	Recall (%)	F1-score (%)	Params (#)	Time (s)	MFLOPs
w/o pruning	98.78	93.69	85.44	89.38	190,738,856	0.0545	7142.88
network-level (Conv+FC)	98.96	**94.65**	88.79	91.63	190,730,981	0.0511	7130.43
block-level (REBs)	98.12	92.60	80.31	86.02	19,118,504	0.0346	714.15
MLPO (ours)	**98.97**	94.63	**89.89**	**92.05**	**19,110,641**	**0.0312**	**713.14**

The bold values indicate the best results among the compared pruning configurations.

In addition, an appropriate pruning ratio can improve model compactness while preserving classification performance. Therefore, additional experiments were conducted to analyze the impact of different pruning ratios on model accuracy and parameter size. As shown in **Table 11**, the classification accuracy remains relatively stable as the pruning ratio increases, while the number of parameters decreases significantly. This phenomenon can be attributed to the removal of redundant low-magnitude weights and the presence of residual connections, which facilitate effective feature reuse and stable information propagation. Among the evaluated configurations, a pruning ratio of 0.9 achieves the best trade-off between classification accuracy and model compactness for HPRNet.

**Table 11:** Analysis of different pruning ratio (*r*) of L1-norm-based unstructured pruning strategy on the model’s classification accuracy and parameter number.

Pruning ratio	*r* = 0.1	*r* = 0.2	*r* = 0.3	*r* = 0.4	*r* = 0.5	*r* = 0.6	*r* = 0.7	*r* = 0.8	*r* = 0.9
Accuracy (%)	98.78	98.96	98.88	98.90	98.82	98.86	98.84	98.86	98.97
Params (#)	171,669,055	152,599,254	133,529,450	114,459,649	95,389,848	76,320,047	57,250,246	38,180,442	19,110,641

**Figure 10:**
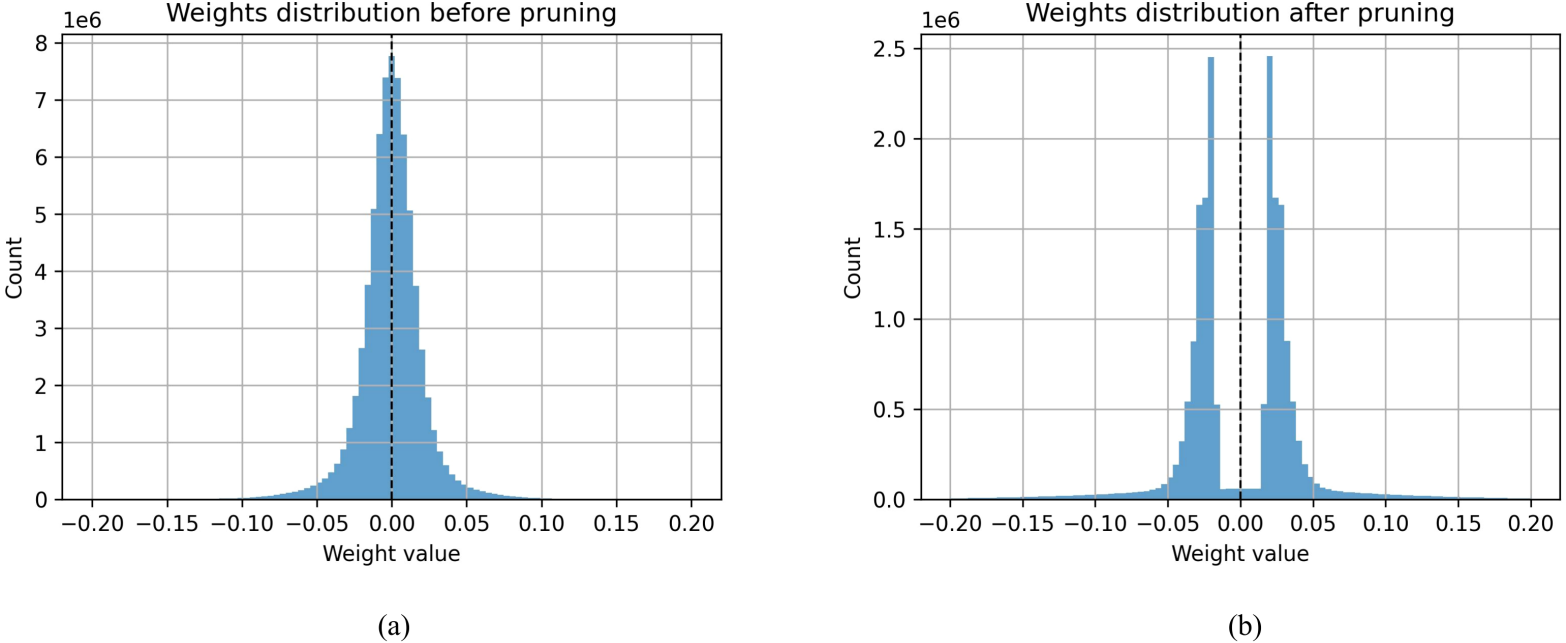
Comparison of parameter distribution before and after pruning. **(a)** Weights distribution before pruning. **(b)** Weights distribution after pruning.

To further illustrate the effect of MLPO, **Figures 10A, B** present the weight distributions of HPRNet before and after pruning with a ratio of 0.9. Before pruning, a large proportion of weights have small absolute values and contribute marginally to classification performance. After pruning, the weight distribution becomes significantly sparser, indicating that many redundant weights have been removed. These results further confirm that the proposed MLPO strategy effectively produces a more compact model while preserving critical weight structures for accurate arrhythmia classification.

### 4.7 Limitations

While the proposed HPRNet framework demonstrates encouraging classification performance, several limitations remain. First, this study does not explicitly incorporate specialized mechanisms designed for morphologically similar ECG categories, whose subtle differences may increase classification difficulty. Future work will explore more discriminative feature learning or targeted optimization strategies, such as attention mechanisms and contrastive representation learning Ma et al. (2026) to improve recognition of similar categories. Second, heartbeat categories in ECG datasets are often highly imbalanced Zhao et al. (2025, 2026). Future work will explore strategies such as data augmentation, class-balanced training, or cost-sensitive learning to further improve the recognition of underrepresented categories. In addition, this study primarily emphasizes empirical evaluation of the proposed deep learning architecture. A deeper theoretical investigation of the model’s convergence behavior and generalization properties remains an interesting direction for future work.

## 5 Conclusion

This paper proposed HPRNet, a hierarchical pyramidal residual network for multi-class ECG arrhythmia classification. The proposed architecture progressively learns hierarchical ECG representations through stacked residual layers composed of Residual Extraction Blocks (REBs), enabling effective modeling of both waveform morphology and long-term rhythm characteristics. To alleviate parameter redundancy and computational overhead introduced by deep residual structures, a Multi-Level Pruning Optimization (MLPO) strategy was incorporated at both the network and block levels, which effectively compresses the model while preserving its discriminative capability. Experimental results on the MIT-BIH and INCART datasets demonstrate that HPRNet achieves competitive classification performance with favorable computational efficiency. Visualization analyses further reveal that the network progressively captures ECG features from local waveform morphology to broader temporal context. Meanwhile, the results also highlight the intrinsic difficulty of distinguishing challenging heartbeat categories, such as S and F. Additional studies on noise robustness and architectural components further validate the effectiveness and stability of the proposed framework. In particular, the HRB and the MLPO strategy contribute to improved feature representation and computational efficiency. Future work will investigate class-balanced learning strategies and inter-patient evaluation protocols to further improve the robustness and generalization capability of the proposed model.

## Data Availability

Publicly available datasets were analyzed in this study. This data can be found here: The datasets analyzed in this study are publicly available from PhysioNet. MIT-BIH Arrhythmia Database: https://physionet.org/content/mitdb/ INCART Database: https://physionet.org/content/incartdb/.
